# Synthesis of High-performance LiNi_0.6_Co_0.2_Mn_0.2_O_2_ Cathode Material for Lithium-ion Batteries by Using a Four Times Liquid Nitrogen Quenching Method and an Al_2_O_3_ Coating Method

**DOI:** 10.3390/ma12223666

**Published:** 2019-11-07

**Authors:** Wenyuan Yang, Yinze Zuo, Qi Chen, Yan Zhang

**Affiliations:** 1 National Laboratory of Solid State Microstructures, Nanjing University, Nanjing 210093, China; yinzezuo@126.com; 2 Yunnan Jingxi New Material Technology Co., Ltd., Qujing 655000, China; chenqi890312@sina.com (Q.C.); cooly713@sina.com (Y.Z.)

**Keywords:** lithium ion batteries, LiNi_0.6_Co_0.2_Mn_0.2_O_2_, liquid nitrogen, Al_2_O_3_, electrochemical performance

## Abstract

Based on the normal co-precipitation method to synthesize LiNi_0.6_Co_0.2_Mn_0.2_O_2_ cathode material, we propose a novel approach using a liquid nitrogen quenching method to synthesize Al_2_O_3_ coated LiNi_0.6_Co_0.2_Mn_0.2_O_2_ cathode material. In the whole process, liquid nitrogen was used four times to quench the materials from high temperatures (50 °C, 750 °C, 90 °C, 500 °C) to −196 °C rapidly in four stages. Various characterizations proved that this method could help to improve the electrochemical performance of lithium-ion batteries. Especially at 5 C rate current, after 100 cycles, the specific discharge capacities were 24.5 mAh/g (LNCM 622), 43.8 mAh/g (LNCM 622-LN), and 53.9 mAh/g (LNCM 622-LN@Al_2_O_3_). Liquid N_2_ quenching increased the charge/discharge capacities and the Al_2_O_3_ layer increased the cycle stability at high current, to finally obtain improved electrochemical properties.

## 1. Introduction

Rechargeable lithium-ion batteries are widely used for energy storage. The nickel-rich layered LiNi_x_Co_y_Mn_1−x−y_O_2_ materials have received significant attention because of their high reversible capacities [1]. Among them, LiNi_0.6_Co_0.2_Mn_0.2_O_2_ is one of the most promising nickel-rich layered cathode materials [2,3], LiNi_0.6_Co_0.2_Mn_0.2_O_2_ is the best compromise between stability and performance aspects, also at elevated temperature [4,5]. Various methods have been applied to synthesize this material, such as the co-precipitation method [6,7] (the composition under homogeneous, synthesis conditions is easy to control), the solid-state method [8] (it is easy to introduce impurities, so the electrochemical performance is not stable), and spray-drying [9] (the uniformity of particles is better). Since the co-precipitation method is widely used in commercial production, we used it in this study to synthesis new materials. However, LiNi_0.6_Co_0.2_Mn_0.2_O_2_ is needed to further improve its structural stability and electrochemical performance [10,11]. In this work, based on the traditional co-precipitation method, we used liquid nitrogen quenching to help synthesize a high-performance LiNi_0.6_Co_0.2_Mn_0.2_O_2_ cathode material.

Quenching samples into liquid nitrogen (−196 °C) is a typical technique to obtain an ultrahigh cooling rate. The cooling rate is uniform and controlled by heat transfer in the quench medium instead of heat conduction in the sample. It is widely used in the preparation of crystals [12], nanomaterials [13] and alloys [14]. Using liquid nitrogen to synthesize cathode materials for lithium-ion batteries has been reported, such as Li_3_V_2_(PO_4_)_3_/C [15], LiFePO_4_/C [16], and LiMnPO_4_/C [17]. According to these reports, the liquid N_2_ quenching method to prepare materials can inhibit the growth and agglomeration of particles, produce some crystal defects, which can expand the tunnels for Li-ion diffusion and shorten the diffusion distance, which are of benefit for the electrochemical performance. However, according to published reports, no one has used liquid nitrogen to help synthesize nickel-rich layered LiNi_x_Co_y_Mn_1−x−y_O_2_ materials; this is the first time that we have used liquid nitrogen quenching method to synthesize LiNi_0.6_Co_0.2_Mn_0.2_O_2_ cathode material. At the same time, using Al_2_O_3_ to coat LiNi_1/3_Co_1/3_Mn_1/3_O_2_ cathode materials has been reported before [18], its electrochemical performances can be improved and the structure is not affected by the coating of Al_2_O_3_. Since the liquid nitrogen quenching method and Al_2_O_3_ coated method are both useful for improving the electrochemical properties of cathode materials, we combined them for the whole process to synthesize high performance LiNi_0.6_Co_0.2_Mn_0.2_O_2_ cathode material.

## 2. Experimental

### 2.1. Preparation of LiNi_0.6_Co_0.2_Mn_0.2_O_2_

First, we synthesized the normal LiNi_0.6_Co_0.2_Mn_0.2_O_2_ powder by the co-precipitation method. (1) NiSO_4_·6H_2_O, CoSO_4_·7H_2_O, and MnSO_4_·H_2_O powders were dissolved in deionized water to obtain 2.0 mol/L solution, with molar ratio of Ni:Co:Mn as 6:2:2. (2) The mixture was heated at 50 °C in a water bath, then NH_3_·H_2_O (2.0 mol/L) and NaOH (2.0 mol/L) were added into the solution to control the pH between 11 and 12 under N_2_ atmosphere. (3) After being stirred for 10 min and filtering the solution, the precipitated powder was washed with deionized water several times and dried at 80 °C in a vacuum oven for 24 h; this was the Ni_0.6_Co_0.2_Mn_0.2_(OH)_2_ precursor. Then a mixture of LiOH and Ni_0.6_Co_0.2_Mn_0.2_(OH)_2_ at a molar ratio of 1.05:1 was heated at 750 °C for 15 h under a flowing oxygen atmosphere, then cooled down slowly from 750 °C to room temperature in the oven (about 5 h), and marked as LNCM 622.

**Figure 1:**
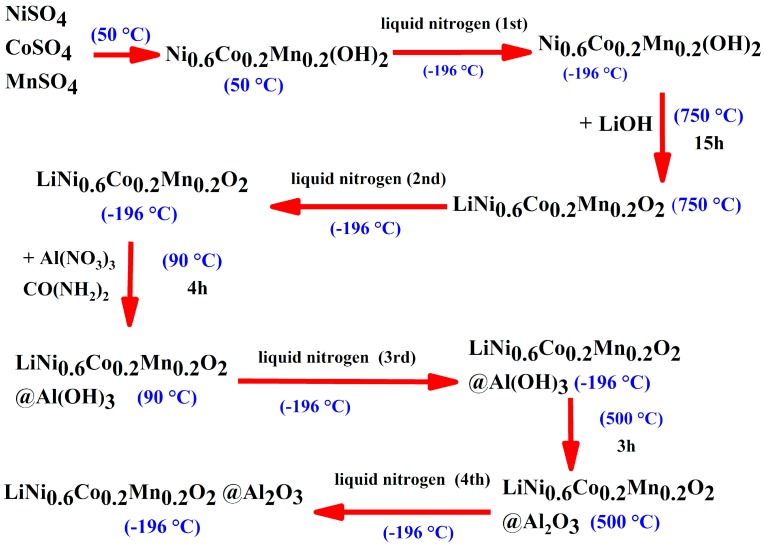
Synthesis method of LNCM 622-LN and LNCM 622-LN@Al_2_O_3_.

**Figure 2:**
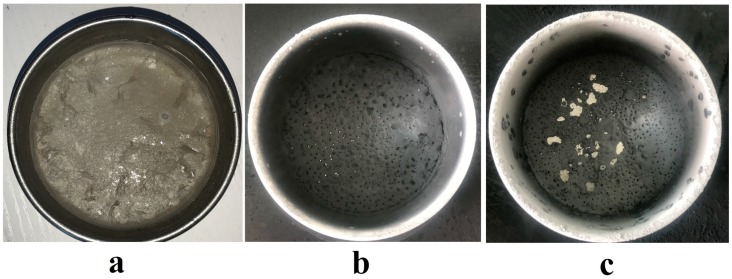
Digital photos of Ni_0.6_Co_0.2_Mn_0.2_(OH)_2_ (**a**), LNCM 622-LN (**b**), LNCM 622-LN@Al_2_O_3_ (**c**).

As a comparison, we synthesized new LiNi_0.6_Co_0.2_Mn_0.2_O_2_ powder with the liquid N_2_ quenching and Al_2_O_3_ coated method. The schematic of the whole process is shown in Figure 1. First, after the Ni_0.6_Co_0.2_Mn_0.2_(OH)_2_ precursor solution was stirred for 10 min, it was poured into liquid nitrogen quickly and the sample cooled down rapidly from 50 °C to −196 °C in 1 min; the 1st liquid nitrogen quenching, for Ni_0.6_Co_0.2_Mn_0.2_(OH)_2_, as shown in Figure 2a. Thirty min later, the precursor powder was washed with deionized water and dried at 80 °C, then used to synthesize LiNi_0.6_Co_0.2_Mn_0.2_O_2_ with LiOH as described above at 750 °C for 15 h. After 15 h, the powder (at 750 °C) was taken out of the oven and immediately added quickly into liquid nitrogen, where it cooled down rapidly from 750 °C to −196 °C in 1 min; the 2nd liquid nitrogen quenching, for LiNi_0.6_Co_0.2_Mn_0.2_O_2_), as shown in Figure 2b, marked as LNCM 622-LN.

To synthesize the 1% Al_2_O_3_ coated LiNi_0.6_Co_0.2_Mn_0.2_O_2_ powder, a specified amount of CO(NH_2_)_2_ was added into deionized water to form a 1.0 mol/L solution. First 10 g LNCM 622-LN and 0.736 g Al(NO_3_)_3_ was added into deionized water and stirred at 90 °C for 30 min before adding into 2.0 mL of CO(NH_2_)_2_ solution and stirring for 4 h. After 4 h, the solution was poured into liquid nitrogen quickly and the temperature cooled down rapidly from 90 °C to −196 °C in 1 min; the 3rd liquid nitrogen quenching, for LiNi_0.6_Co_0.2_Mn_0.2_O_2_@Al(OH)_3_). Sixty min later, the Al(OH)_3_ coated LiNi_0.6_Co_0.2_Mn_0.2_O_2_ powder was washed with deionized water and dried at 80 °C. Then this sample was heated at 500 °C for 3 h. After 3 h was over, the powder (at 500 °C) was taken out of the oven immediately, rapidly poured into liquid nitrogen and cooled down rapidly from 500 °C to −196 °C in 1 min; the 4th liquid nitrogen quenching, for LiNi_0.6_Co_0.2_Mn_0.2_O_2_@Al_2_O_3_). Now we obtained the 1% Al_2_O_3_ coated LiNi_0.6_Co_0.2_Mn_0.2_O_2_ powder, as shown in Figure 2c, marked as LNCM 622-LN@Al_2_O_3_.

### 2.2. Characterization

The coin cells (type 2032) were assembled in a pure argon-filled glove box with the content of H_2_O and O_2_ less than 1 ppm. A mixture of 80 wt.% cathode material, 10 wt.% Super P carbon black and 10 wt.% polyvinylidene fluoride (PVDF) were mixed in N-methylpyrrolidone (NMP) to produce a slurry. The slurry was coated on aluminum foil (150 μm thickness) and dried at 80 °C for 12 h in a vacuum oven. The electrolyte was 1.0 M LiPF6/ EC + DMC + EMC (1:1:1, volume ratio). Celgard 2400 was used as separator. 

A Hitachi S-3400N field emission scanning electron microscope (SEM) was used observing at 30 kV. X-ray diffraction (XRD) powder patterns of the samples were taken on an ARL X’TRA powder diffractometer with Cu-Kα radiation (λ = 0.154 nm). The electrochemical charge/discharge properties were tested by Land CT 2001A (5 V, 50 mA) between 2.8 and 4.2 V (vs. Li/Li^+^) at different rates (1 C = 276.55 mAh/g). All the cells were charged and discharged at 0.05 C for the first 5 cycles, then a constant current was used (0.1 C, 0.5 C, 1 C, 2 C, 5 C and 10 C, separately) charged until the voltage >4.2 V, next charged at constant voltage (4.2 V) with a taper current, cut off until the taper current was less than 10% of the above constant current (less than 0.01 C, 0.05 C, 0.1 C, 0.2 C, 0.5 C and 1 C, separately). After that, the used constant current was discharged until the voltage <2.8 V. The above cycle was repeated 50 times (0.1 C) or 100 times (other rates).

## 3. Results and Discussion

**Figure 3:**
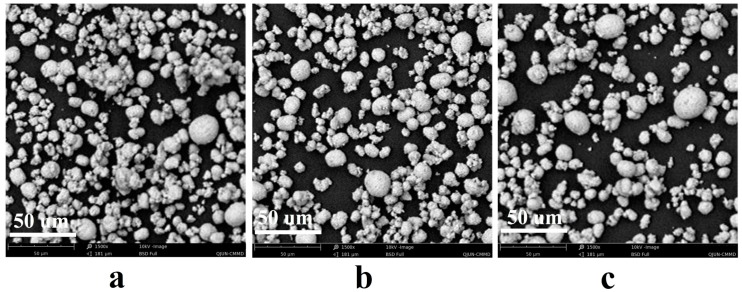
SEM images of LNCM 622 (**a**), LNCM 622-LN (**b**), and LNCM 622-LN@Al_2_O_3_ (**c**).

Figure 3 shows the SEM images of LNCM 622, LNCM-622LN and LNCM 622-LN@Al_2_O_3_, the particle size is about 10–20 μm in diameter. Compared with LNCM 622, the particles of LNCM 622-LN and LNCM 622-LN@Al_2_O_3_ are less aggregated, with more spherical morphology, but the differences are small, showing that liquid nitrogen helped the materials form more regular shapes to some extent. 

**Figure 4:**
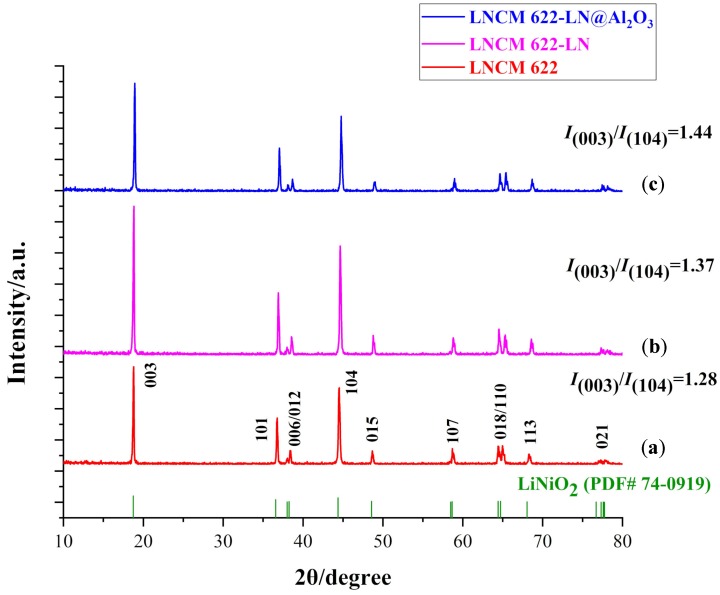
XRD patterns of LNCM 622 (**a**), LNCM 622-LN (**b**), and LNCM 622-LN@Al_2_O_3_ (**c**).

Figure 4 displays the XRD patterns of the three samples. All diffraction peaks are indexed based on the α–NaFeO_2_ structure (space group: *R*3m). All the XRD patterns show clear splits between the (006/012) and (108/110) peaks, especially for LNCM 622-LN and LNCM 622-LN@Al_2_O_3_, where the splits are clearer, meaning that the materials have a typical hexagonal structure [19,20]. Since the Al_2_O_3_ layer is amorphous and the content is very low, no peak of the Al_2_O_3_ crystal type is observed.

The intensity ratio of *I*_(003)_/*I*_(104)_ characterizes the degree of cation mixing of the cathode material. If *I*_(003)_/*I*_(104)_ <1.2, indicates a high degree of cation mixing, due primarily to the occupancy of other ions in the lithium region [20,21]. The higher the ratio, the lower is the level of cation mixing, which is a benefit for lithium-ion transfer. The ratio of *I*_(003)_/*I*_(104)_ increases from 1.28 (LNCM 622) to 1.37 (LNCM 622-LN) and to 1.44 (LNCM 622-LN@Al_2_O_3_). Especially the ratios of LNCM 622-LN and LNCM 622-LN@Al_2_O_3_ are greater than 1.2, showing the synthesized powders have well-ordered structures with no obvious cation mixing. This indicates that liquid N_2_ quenching helped to improve the structural properties of the nickel-rich layered cathode materials.

**Figure 5:**
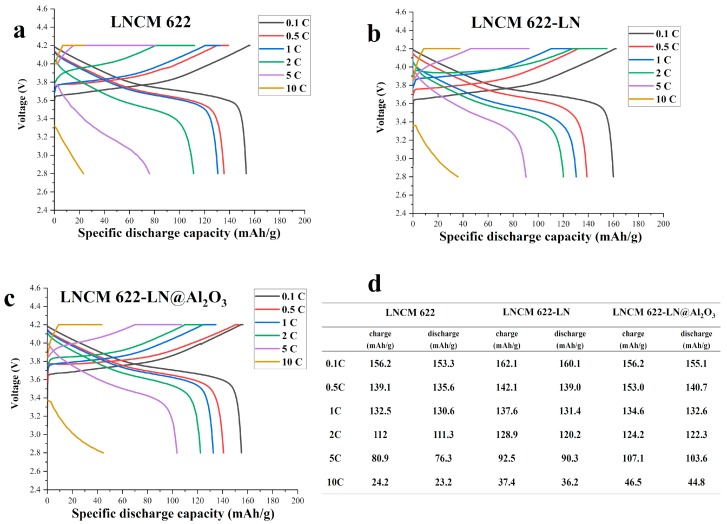
The initial specific charge/discharge curves of LNCM 622 (**a**), LNCM 622-LN (**b**), LNCM 622-LN@Al_2_O_3_ (**c**), and initial charge/discharge capacity data of the 3 samples (**d**).

The initial specific charge/discharge curves of the three samples between 2.8 and 4.2 V are shown in Figure 5. The initial specific discharge capacities at 0.1 C/0.5 C/1 C/2 C/5 C/10 C are 153.3/135.6/130.6/111.3/76.3/23.2 mAh/g (LNCM 622), 160.1/139.0/131.4/120.2/90.3/36.2 mAh/g (LNCM 622-LN) and 155.1/140.7/132.6/122.3/103.6/44.8 mAh/g (LNCM 622-LN@Al_2_O_3_), respectively. At low rate current (0.1 C), the specific discharge capacity of LNCM 622-LN is higher than the other two. However, when the rate current is higher than 0.5 C, LNCM 622-LN@Al_2_O_3_ performs the best of all and in any case, the specific discharge capacity of LNCM 622-LN is always higher than that of LNCM 622. This phenomenon may be due to the material having more tunnels for Li-ions diffusion, and the diffusion distance being shorter, indicating that liquid N_2_ quenching could help to increase the material’s electrochemical performance, similar to the work referenced above [15,16,17]. Although Al_2_O_3_ does not participate in electrochemical reactions, it could improve the cycle stability at a high rate current, so the discharge capacity of LNCM 622-LN@Al_2_O_3_ is lower at 0.1 C, but exhibits the highest value at the strong rate currents (0.5 C, 1 C, 2 C, 5 C and 10 C).

**Figure 6:**
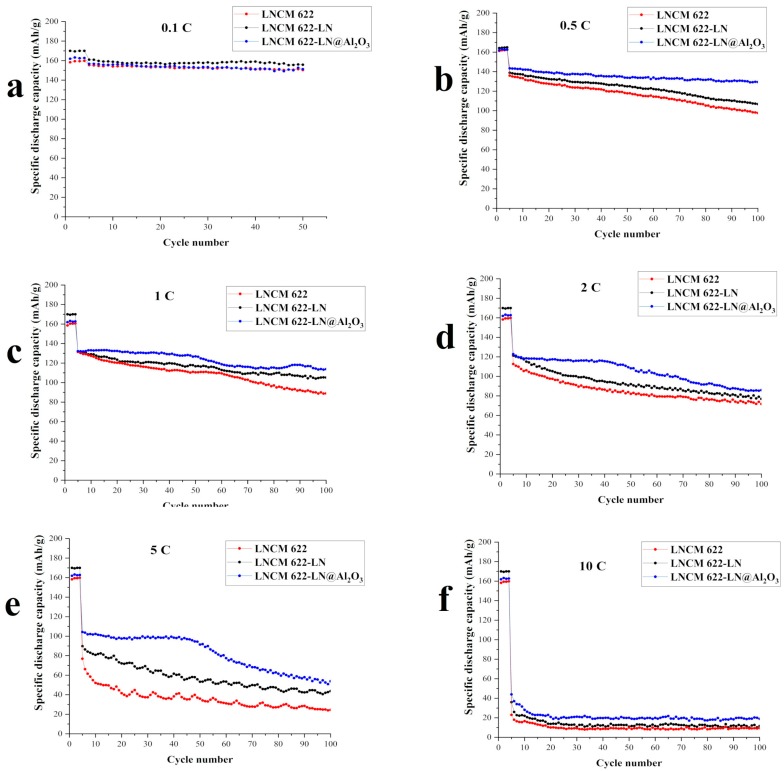
Cycling performance of LNCM 622, LNCM 622-LN and LNCM 622-LN@Al_2_O_3_ at 0.1 C (**a**), 0.5 C (**b**), 1 C (**c**), 2 C (**d**), 5 C (**e**), and 10 C (**f**).

Figure 6 shows the cycling performance of the three samples at different rate currents between 2.8 and 4.2 V. At all rates, in the last cycle, compared with LNCM 622, the specific discharge capacities of LNCM 622-LN and LNCM 622-LN@Al_2_O_3_ are significantly higher, the increased proportions are about 3.66% and 0.67% (0.1 C), 9.32% and 32.58% (0.5 C), 18.36% and 28.04% (1 C), 7.25% and 19.80% (2 C), 78.78% and 120.00% (5 C), 23.40% and 101.06% (10 C), respectively. Especially at the 5 C and 10 C rate, the specific discharge capacities’ values almost doubled. This means that, for LNCM 622-LN, liquid N_2_ quenching increased the discharge capacities. Also for LNCM 622-LN@Al_2_O_3_, the increase of discharge capacities was not just due to the use of liquid N_2_, the Al_2_O_3_ layer also improved the cycle stability of LiNi_0.6_Co_0.2_Mn_0.2_O_2_ at high currents, finally improving the cycling performance.

**Figure 7:**
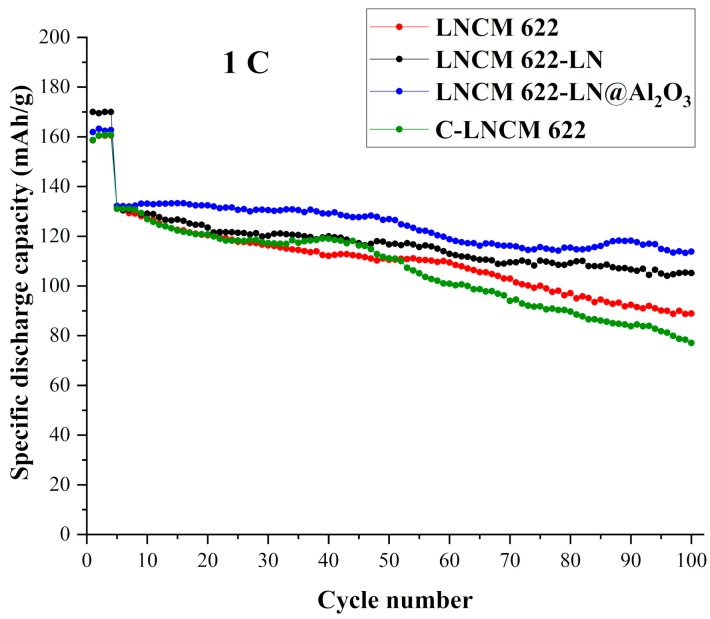
Cycling performance of LNCM 622, LNCM 622-LN, LNCM 622-LN@Al_2_O_3_, and C-LNCM 622 at 1 C.

In addition, it was worthwhile to compare these materials with commercial materials. The commercial LiNi_0.6_Co_0.2_Mn_0.2_O_2_ cathode material was obtained from Shenzhen Kejing Star Technology Co., Ltd. (Shenzhen, China), marked as C-LNCM 622. We prepared cells and tested this material under the same conditions as mentioned above. As shown in Figure 7, at 1 C rate, the initial specific discharge capacity of C-LNCM 622 is very close to our synthesized materials. In the 30th to 50th cycle, its value was higher than LNCM 622, nearly equal to LNCM 622-LN, but lower than LNCM 622-LN@Al_2_O_3_. However, in the final cycle, the specific discharge capacity of C-LNCM 622 was lower than our synthesized samples. Every method has its advantages or disadvantages and compared with LNCM 622, the commercial material has a better performance in the middle cycles. However, the liquid N_2_ quenching and Al_2_O_3_ coated method also demonstrate many advantages in improving cycling performance.

## 4. Conclusions

Using liquid N_2_ to rapidly quench LiNi_0.6_Co_0.2_Mn_0.2_O_2_ powder and Al_2_O_3_ coated powder from high temperatures (50 °C, 750 °C, 90 °C, 500 °C) to −196 °C was found to be an efficient method to improve the electrochemical performance of lithium-ion batteries. In this work, we used the liquid N_2_ quenching method four times over four stages, in order to make new cathode materials with better electrochemical properties, with a coated Al_2_O_3_ layer to obtain a better cycle stability at high currents. Compared with normal LNCM 622 and commercial LiNi_0.6_Co_0.2_Mn_0.2_O_2_ cathode material, LNCM 622-LN and LNCM 622-LN@Al_2_O_3_ have a better ordered structure with less cation mixing, higher initial charge/discharge capacities, and better cycling performance at different rates of currents. Liquid nitrogen is easy to prepare, volatilizes quickly without residue giving no pollution to the environment, and liquid N_2_ quenching is a mature technology in industrial production. Therefore, this is a promising method to synthesize many other high-performance nickel-rich layered cathode materials like LiNi_0.8_Co_0.1_Mn_0.1_O_2_, LiNi_0.5_Co_0.2_Mn_0.3_O_2_, LiNi_0.33_Co_0.33_Mn_0.33_O_2,_ and LiNi_0.85_Co_0.1_Mn_0.05_O_2_. All the above materials could also be coated with Al_2_O_3_ layer by this method. We believe these cathode materials also demonstrate outstanding electrochemical properties.
